# Shikimic acid attenuates oxidative stress-induced senescence in 3D4/21 cells by modulating PARP1 activity and DNA damage response

**DOI:** 10.3389/fnut.2025.1614148

**Published:** 2025-11-27

**Authors:** Kaibin Mo, Chengyu Wu, Weichun Wu, Yicheng Zhang, Linlin Yang, Li Li, Xianhui Huang

**Affiliations:** 1 Guangdong Key Laboratory for Veterinary Drug Development and Safety Evaluation, College of Veterinary Medicine, South China Agricultural University, Guangzhou, China; 2 College of Animal Science, South China Agricultural University, Guangzhou, China

**Keywords:** shikimic acid, oxidative stress, 3D4/21 cells, cellular senescence, inflammation, transcriptomics

## Abstract

**Background:**

Alveolar macrophage (AM) dysfunction driven by oxidative stress contributes significantly to human chronic lung diseases like COPD and IPF. This oxidative stress often leads to DNA damage and cellular senescence, perpetuating inflammation. Shikimic acid (SA), a natural compound with antioxidant potential, requires investigation for its specific protective mechanisms in AMs, particularly regarding DNA repair and senescence modulation, to evaluate its therapeutic relevance.

**Methods:**

Porcine AMs (3D4/21 cells) were pretreated with SA and the positive control N-Acetyl-L-cysteine, respectively, before oxidative challenge with tert-butyl hydroperoxide (TBHP). We assessed cell viability (CCK-8), cytotoxicity (LDH), oxidative stress and inflammation markers (ROS, NO, iNOS, COX-2). Transcriptomics identified global gene/pathway changes. Western blotting validated key DNA damage response (DDR) proteins (XRCC1 and PARP1) and poly (ADP-ribose) (PAR) levels (indicative of PARP1 activity). Senescence was determined via senescence-associated β-galactosidase (SA-β-gal) staining and ELISA for senescence-associated secretory phenotype (SASP) factors (TNF-α, IL-1β, IL-6, IL-8).

**Results:**

SA pretreatment significantly enhanced cell viability, decreased LDH release (*P* < 0.05), and markedly reduced intracellular ROS and NO levels (*P* < 0.05) compared to TBHP-stressed controls. Pro-inflammatory iNOS and COX-2 expression was also significantly lowered by SA (*P* < 0.05). Transcriptomics revealed significant alterations in gene expression, highlighting enrichment in DNA repair and senescence pathways. Mechanistically, SA significantly counteracted the TBHP-induced upregulation of XRCC1 and PARP1 protein levels, and PAR levels (*P* < 0.05), suggesting normalization of the DDR. Furthermore, SA substantially decreased the percentage of senescent (SA-β-gal positive) cells (*P* < 0.05) and suppressed the secretion of multiple SASP factors (*P* < 0.05).

**Conclusion:**

SA effectively restores homeostasis in oxidatively injured 3D4/21 cells by concurrently mitigating oxidative stress and inflammation, modulating the DDR, particularly PARP1 activation and associated factors, and attenuating cellular senescence. This positions SA as a promising candidate for further investigation as a therapeutic agent for human lung diseases characterized by macrophage dysfunction and oxidative pathology.

## 1 Introduction

Alveolar macrophages (AMs) are essential immune sentinels for maintaining lung health, orchestrating immune defense and tissue homeostasis (1). However, it has been demonstrated that chronic exposure to oxidative stress significantly impairs the function of AMs, driving inflammation and tissue damage characteristic of prevalent human lung diseases like Chronic Obstructive Pulmonary Disease (COPD) and Idiopathic Pulmonary Fibrosis (IPF) (2, 3). Consequently, the effective protection of AMs from oxidative injury is a critical therapeutic objective.

Indeed, persistent oxidative stress inflicts substantial DNA damage within AMs (4). Failure to efficiently repair such damage through pathways like base excision repair (BER) can lead to persistent DNA damage response (DDR) signaling (5). However, chronic damage or dysregulated repair processes can trigger premature cellular senescence (6, 7). Senescent AMs contribute to lung pathology by secreting a pro-inflammatory senescence-associated secretory phenotype (SASP), fueling a cycle of chronic inflammation detrimental to tissue resolution (8, 9). Consequently, interventions that mitigate oxidative stress-induced DNA damage and prevent pathological senescence in AMs are highly sought after.

Shikimic acid (SA), a naturally occurring cyclohexene derivative, is present in high concentrations in *Illicium verum* (star anise) and is widely recognized as a key precursor for the antiviral drug oseltamivir (10). Beyond this role, accumulating evidence suggests that SA possesses intrinsic biological activities, including significant antioxidant and anti-inflammatory properties, as demonstrated in various *in vitro* and *in vivo* models (11, 12). Despite its known bioactivities, the precise mechanisms by which SA modulates the complex DDR—particularly the critical PARP1 signaling axis—and prevents senescence specifically in oxidatively challenged AMs remain largely unexplored. Understanding these mechanisms is crucial for evaluating SA's potential therapeutic application in human respiratory diseases. Therefore, this study aimed to characterize SA's protective effects in a porcine AM cell line (3D4/21) under tert-butyl hydroperoxide (TBHP)-induced oxidative stress, focusing on its impact on PARP1 activation, associated DDR components, and downstream cellular senescence pathways.

## 2 Materials and methods

### 2.1 Cells and compounds

The 3D4/21 cells were obtained from the China Center for Type Culture Collection (CCTCC; Wuhan, China), catalog number GDC0755. SA with a purity of ≥99% was purchased from FuKang Pharmaceutical Co., Ltd (Fuzhou, China). A 100 mM stock solution of SA was prepared by dissolving the compound in sterile deionized water. N-Acetyl-L-cysteine (NAC) and TBHP were acquired from AbMole Co., Ltd (Shanghai, China), with product codes AMB23156 and AMB19872, respectively. Unless indicated otherwise, all culture reagents were filter-sterilized using 0.22 μm polyether sulfone membranes (Biosharp, BS-PES-22, Hefei, China).

### 2.2 Cell experiments

#### 2.2.1 Cell culture

The 3D4/21 cells were cultivated in Roswell Park Memorial Institute (RPMI) 1640 medium (Gibco, 12633020, New York, USA) with the addition of 10% fetal bovine serum (LONSERA, S711-001S, Suzhou, China), 100 IU/mL penicillin, and 100 μg/ml streptomycin (Pricella, PB180120, Wuhan, China). Standard culture conditions comprised incubation at 37 °C in a humidified atmosphere containing 5% CO_2_.

#### 2.2.2 Cell viability assay

3D4/21 cells were seeded in 96-well plates at a density of 2 × 10^4^ cells/well and allowed to adhere for 24 h. Thereafter, the medium was then replaced with RPMI 1,640 medium containing SA at concentrations of 0, 2, 10, 50, 250, and 1,250 μM. Following a 12 h incubation period, cell viability was quantified using the Cell Counting Kit-8 (CCK-8; AbMole, M4839, Shanghai, China) assay according to the manufacturer's instructions. Control wells (cells only) and blank wells (RPMI 1640 medium only) were included. Absorbance was measured at 450 nm with a reference wavelength of 650 nm using a microplate reader (Thermo Fisher Scientific, Multiskan FC, Massachusetts, USA). Cell viability was thus calculated as follows:

Cell viability = (Test well absorbance – Blank well absorbance)/(Control well absorbance – Blank well absorbance).

#### 2.2.3 Oxidative stress induction

3D4/21 cells seeded in 96-well plates were subjected to TBHP at final concentrations of 0, 1, 5, 10 and 15 μM. Oxidative stress was induced for 15 min and 30 min, after which cell viability was quantified using the CCK-8 assay, according to the manufacturer's protocol. Based on dose-response analysis, the TBHP concentration and exposure duration that resulted in approximately 50% cell viability reduction were selected as the parameters for subsequent oxidative stress modeling (13).

#### 2.2.4 Experimental groups and treatments

In light of the cell viability results and the reported effective concentrations of SA in other *in vitro* studies, concentrations of 10, 50, and 250 μM, representing approximate half-log intervals, were selected for the purpose of evaluating dose-dependent effects (11, 14). In the course of the experimental setup, the 3D4/21 cells were methodically allocated into six distinct treatment groups: (1) negative control (Ctrl: RPMI 1640 medium only); (2) oxidative stress induction group (TBHP: TBHP treatment); (3) antioxidant control (NAC: 10 mM NAC pretreatment for 12 h); (4–6) SA intervention groups (SA10: 10 μM, SA50: 50 μM, SA250: 250 μM SA pretreatment for 12 h). A comprehensive summary of the experimental parameters is provided in Table 1. These parameters include the duration of treatment, the concentrations of reagents, and the sequences in which the reagents were administered.

**Table 1:** Experimental groups and treatments.

**Groups**	**Treatments**
Ctrl	RPMI 1640 medium incubation for 12 h
TBHP	TBHP induced oxidative stress after 12 h of incubation in RPMI 1640 medium
NAC	TBHP induced oxidative stress after 12 h of incubation in RPMI 1640 containing 10 mM NAC
SA10	TBHP induced oxidative stress after 12 h of incubation in RPMI 1640 containing 10 μM SA
SA50	TBHP induced oxidative stress after 12 h of incubation in RPMI 1640 containing 50 μM SA
SA250	TBHP induced oxidative stress after 12 h of incubation in RPMI 1640 containing 250 μM SA

#### 2.2.5 Cytotoxicity assay

3D4/21 cells were inoculated in 96-well plates and six experimental groups were set up according to Table 1. The content of lactate dehydrogenase (LDH) in the medium was determined by means of a commercial kit (Beyotime, C0016, Shanghai, China), and cell viability was assessed by CCK8 assay.

#### 2.2.6 Reactive oxygen species (ROS) measurement

3D4/21 cells were seeded in 6-well plates at 1 × 10^6^ cells/well and allowed to adhere for 24 h under standard conditions. Six experimental groups were established according to Table 1. Intracellular ROS levels were quantified using the ROS Assay Kit (Beyotime, S0033S, Shanghai, China). Cells were incubated with 10 μM DCFH-DA probe for 30 min at 37 °C in the dark, followed by three washes with phosphate-buffered saline (PBS) to remove residual dye. Stained cells were immediately analyzed using an inverted fluorescence microscope (Leica, DMi8, Wetzlar, Germany) equipped with FITC filters (excitation/emission: 488/525 nm). Six randomly selected fields per well were captured at 100 × magnification, and the mean fluorescence intensity (MFI) was quantified.

#### 2.2.7 Measurement of nitric oxide (NO), inducible nitric oxide synthase (iNOS) and cyclooxygenase-2 (COX-2)

3D4/21 cells were seeded in 96-well plates, and six experimental groups were established according to Table 1. The measurement of NO levels in the medium was conducted using commercially available kits (Beyotime, S0021S, Shanghai, China). Subsequent to this, the cells were lysed using RIPA lysis buffer (Beyotime, P0013B, Shanghai, China), following which the lysate was subjected to centrifugation at 12,000 g for 15 min at 4 °C. The resulting supernatant was then analyzed using ELISA kits (Jiangsu Meimian Industrial Co., Ltd, Yancheng, China) to determine the total intracellular protein levels of iNOS and COX-2 in the cell lysates.

### 2.3 RNA sequencing (RNA-seq) and differential gene enrichment analysis

Following the completion of the initial assays (cell viability, LDH, ROS, NO, iNOS, and COX-2), it was determined that the 250 μM SA concentration (SA group) would be utilized for the subsequent transcriptomic analyses, as it exhibited the most significant and substantial protective effects. 3D4/21 cells, which had been seeded in six-well plates, were divided into three groups: Ctrl, TBHP and SA groups, according to the treatments in Table 1, with three replicates in each group, for transcriptomics sequencing analysis.

#### 2.3.1 RNA extraction and library construction

Total RNA was extracted using the TRIzol method, following the manufacturer's instructions. The purity and integrity of the RNA were then assessed using a NanoDrop 2000 spectrophotometer (Thermo Fisher Scientific, Massachusetts, USA) and an Agilent 2100 Bioanalyzer (Agilent Technologies, California, USA). To enrich and fragment the mRNA, OligodT magnetic beads (Thermo Fisher Scientific, 61002, Massachusetts, USA) were used. Reverse transcription was carried out, and the end-repaired and ligated products were then amplified using specific primers to construct a cDNA library for sequencing.

#### 2.3.2 High-throughput sequencing

Libraries were subjected to sequencing using the Illumina NovaSeq 6000 platform, thereby generating 150 bp paired-end reads. Subsequently, we processed raw reads in fastq format to remove those of low quality, thus obtaining clean reads for subsequent analysis. Subsequent to this, the reads were aligned to the reference genome, and gene expression was quantified as read counts per gene. Principal component analysis (PCA) was performed using R (v3.2.0) to assess sample biological replicates.

#### 2.3.3 Differentially expressed genes (DEGs) enrichment analysis

We identified DEGs using the R software DESeq2 (v1.42.0) according to a standardized procedure (15), with thresholds of |log_2_FoldChange| ≥ 1 and padj ≤ 0.05. Gene Ontology (GO) and Kyoto Encyclopedia of Genes and Genomes (KEGG) enrichment analyses were performed using the R software ClusterProfiler (v4.8.1) to determine the biological functions and signaling pathways of DEGs. Finally, the results were visualized using the bioinformatics analysis platform (https://magic-plus.novogene.com).

### 2.4 Experimental validation

#### 2.4.1 Western blot analysis

To validate the transcriptomic analysis results, subsequent Western blot analysis will focus on the Ctrl, TBHP, and SA groups. Total protein was extracted from 3D4/21 cells in the aforementioned treatment groups using RIPA lysis buffer containing protease and phosphatase inhibitors. Protein concentration was determined by the BCA method and normalized accordingly. Protein samples were separated by 10% sodium dodecyl sulfate-polyacrylamide gel electrophoresis and transferred to polyvinylidene difluoride membranes. Membranes were blocked with 5% skimmed milk for 2 h at room temperature. Membranes were then incubated with primary antibodies against XRCC1 (Abcam, ab9147, Cambridge, UK), PARP1 (Affinity Biosciences, DF7198, Cincinnati, USA), and PAR (Cell Signaling Technology, CST 83732, Massachusetts, USA) at 4 °C for 12 h. Subsequently, membranes were incubated with appropriate secondary antibodies for 2 h at room temperature. Protein bands were visualized using ECL chemiluminescence, and images were acquired using a gel imaging system (Azure Biosystems, Azure 400, California, USA). The gray value of protein bands was quantified using ImageJ software, and protein levels were quantified relative β-actin (Proteintech, 66009-1-Ig, Rosemont, USA) as an internal reference.

#### 2.4.2 Measurement of SASP factors

3D4/21 cells were cultured in 96-well plates and divided into three groups (Ctrl, TBHP, and SA according to the treatments in Table 1. The concentrations of tumor necrosis factor (TNF)-α, interleukin (IL)-1β, IL-6, and IL-8 in the culture fluid were measured using ELISA kits (Jiangsu Meiman Industrial Co., Ltd., Yancheng, China) according to the manufacturer's instructions.

#### 2.4.3 Senescence-associated β-galactosidase (SA-β-gal) staining

3D4/21 cells cultured in 6-well plates [Ctrl, TBHP, and SA groups] were fixed with the provided fixative for 15 min at room temperature according to the instructions of the SA-β-gal staining kit (Beyotime, C0602, Shanghai, China). Cells were rinsed three times with PBS. SA-β-gal staining solution was added to the cells. Cells were incubated at 37 °C in the absence of CO_2_ for 12 h. Cell staining was observed using an inverted light microscope. Six randomly selected fields of view per well were imaged at 200 × magnification. The total number of cells and the number of SA-β-gal-positive cells were counted to calculate the percentage of positive cells.

### 2.5 Statistical analysis

One-way ANOVA and Tukey's Multiple Comparison Test were performed using Graph Pad Prism 10 with simultaneous image visualization. Data normality and homogeneity of variances were assessed using Shapiro–Wilk and Levene's tests, respectively, prior to ANOVA. Data are presented as mean ± SD. Specific *P*-values are indicated in the figures, with *P* < 0.05 considered statistically significant.

## 3 Results

### 3.1 Effect of shikimic acid on 3D4/21 cell viability and modeling of oxidative stress

**Figure 1:**
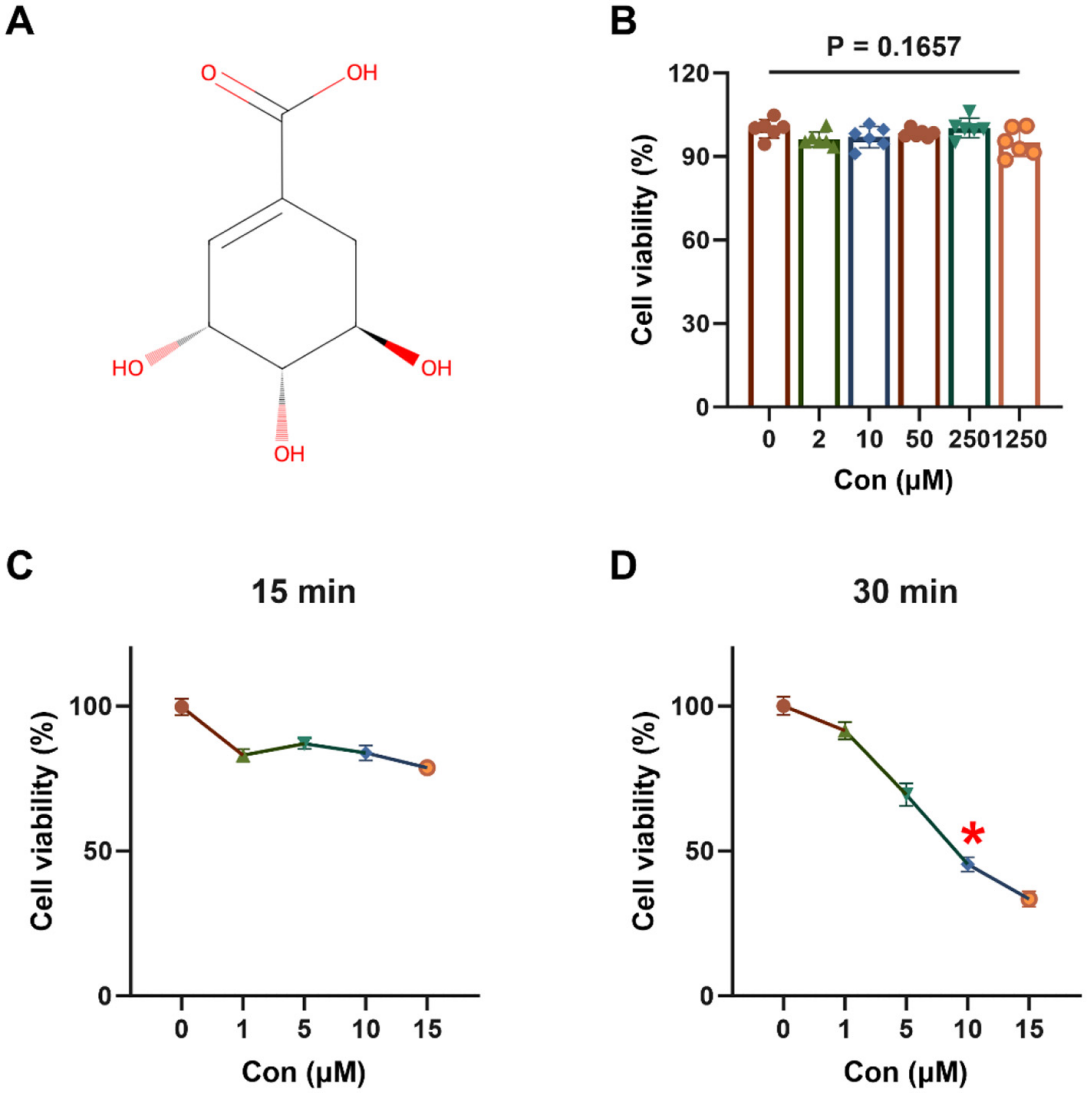
Effect of shikimic acid on 3D4/21 cell viability and modeling of oxidative stress (*n* = 6). **(A)** Chemical structural formula of SA. **(B)** Effect of SA on 3D4/21 cell viability. **(C)** Viability of 3D4/21 cells treated with TBHP for 15 min. **(D)** Viability of 3D4/21 cells treated with TBHP for 30 min. The asterisk (*) indicates that the experimental conditions yielding optimal modeling results correspond to a point where cell viability declines to approximately 50%.

The chemical structure of shikimic acid is illustrated in Figure 1A. Shikimic acid, at concentrations up to 1,250 μM, did not significantly affect the viability of 3D4/21 cells after 12 h incubation (*P* = 0.1657, Figure 1B).

As demonstrated in Figures 1C, D, 3D4/21 cells exposed to TBHP for 15 or 30 min exhibited a decline in cell viability, suggesting the occurrence of oxidative damage. At 15 min, cell viability in all TBHP treatment groups was >50%. However, when the duration of treatment was extended to 30 min, a significant decrease in cell viability was observed, which followed a dose-dependent response. After 30 min of treatment with 10 μM TBHP, cell viability was approximately 45.44%. Consequently, 10 μM TBHP for 30 min was selected as the optimal condition for subsequent oxidative stress modeling.

### 3.2 Effect of shikimic acid on 3D4/21 cell viability and ROS production under oxidative stress

**Figure 2:**
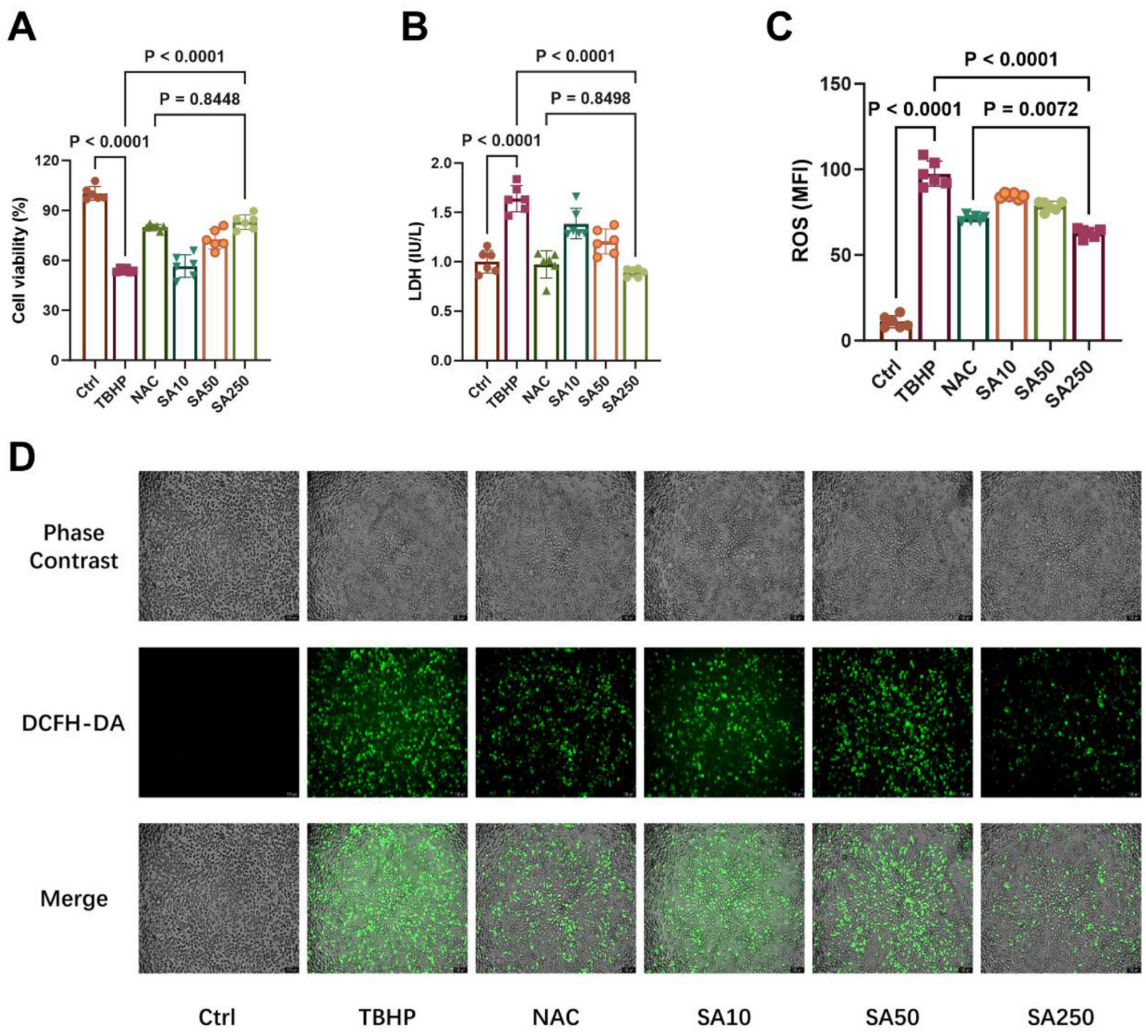
Effect of shikimic acid on 3D4/21 cell viability and ROS production under oxidative stress (*n* = 6). **(A)** Effect of SA on the viability of 3D4/21 cells under oxidative stress. **(B)** Effect of SA on LDH production in 3D4/21 cells. **(C)** Effect of SA on ROS production in 3D4/21 cells. **(D)** Representative ROS fluorescence images of each treatment group. Scale bar = 100 μm, 100 × magnification.

The cell viability of 3D4/21 cells is illustrated in Figure 2A. In comparison with the TBHP group, 50 and 250 μM SA increased cell viability in a dose-dependent manner (*P* < 0.05). The efficacy of 250 μM SA in increasing cell viability was found to be similar to that of 10 mM NAC (*P* = 0.8448).

TBHP treatment considerably elevated LDH release compared to the Ctrl group (*P* < 0.05, Figure 2B). SA, at concentrations of 10, 50, and 250 μM, exhibited a concentration-dependent inhibition of LDH release in comparison with the TBHP group (*P* < 0.05). In addition, no significant difference in LDH content in the medium was observed between the SA250 group and the NAC group (*P* = 0.8498).

As demonstrated in Figure 2C, there was a significant increase in ROS production in 3D4/21 cells in the TBHP group in comparison with the Ctrl group (*P* < 0.05). Conversely, SA, at concentrations of 10, 50, and 250 μM, exhibited a concentration-dependent inhibition of ROS production in comparison to the TBHP group (*P* < 0.05). Furthermore, Figure 2D demonstrates that the SA250 group exhibited a marked decrease in ROS production in comparison to the 10 mM NAC group (*P* = 0.0072).

### 3.3 Effect of shikimic acid on NO, COX-2 and iNOS content under oxidative stress

**Figure 3:**
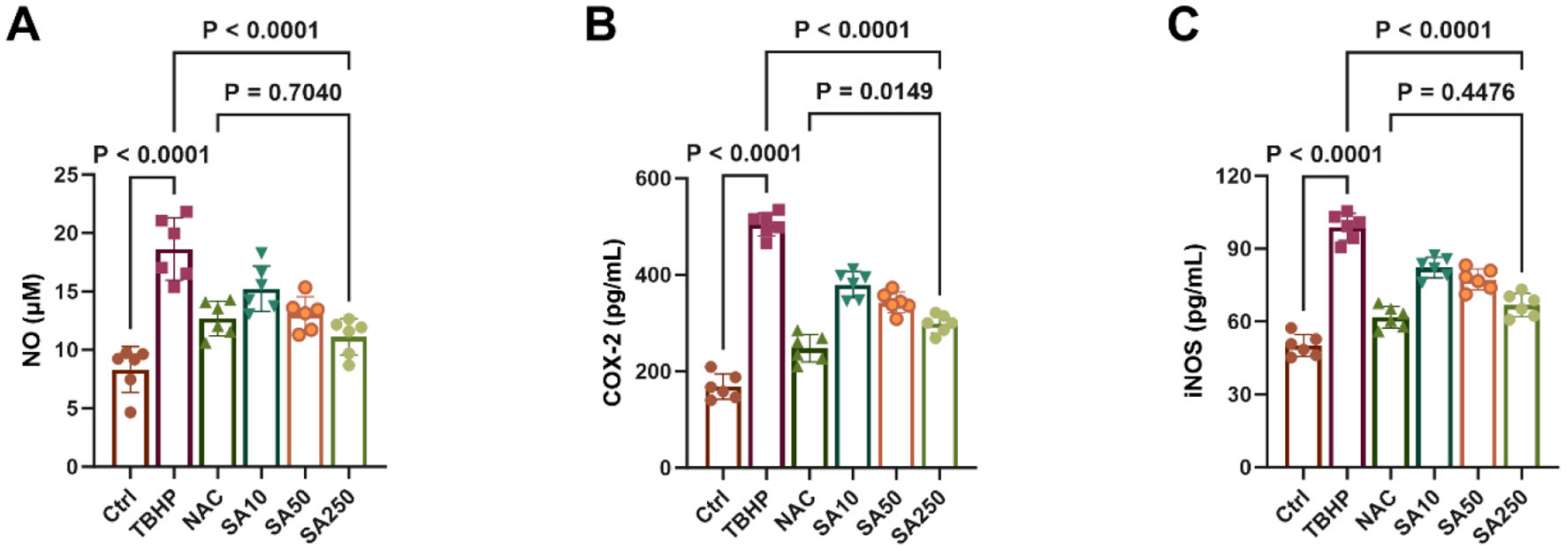
Effect of shikimic acid on NO, COX-2 and iNOS content under oxidative stress (*n* = 6). **(A)** Effect of SA on NO content in 3D4/21 cells. **(B)** Effect of SA on COX-2 content in 3D4/21 cells. **(C)** Effect of SA on iNOS content in 3D4/21 cells.

A significant increase in NO release from 3D4/21 cells was observed in the TBHP group compared to the Ctrl group (*P* < 0.05, Figure 3A). However, the results demonstrate that SA, at concentrations of 10, 50, and 250 μM, exhibited a concentration-dependent inhibition of NO release in comparison with the TBHP group (*P* < 0.05). In addition, a lack of statistically significant difference in NO content in the medium was observed between the SA250 group and the NAC group (*P* = 0.7040).

The content of COX-2 and iNOS in 3D4/21 cells is demonstrated in Figures 3B, C. Treatment of 3D4/21 cells with TBHP resulted in a significant increase in the expression of both enzymes in comparison with the Ctrl group (*P* < 0.05). Conversely, their expression was found to decrease significantly in a dose-dependent manner with increasing SA concentration (*P* < 0.05). It is noteworthy that there was no statistically significant difference in iNOS levels between the SA250 group and the NAC group (*P* = 0.4476).

### 3.4 RNA-seq and differential gene expression analysis

**Figure 4:**
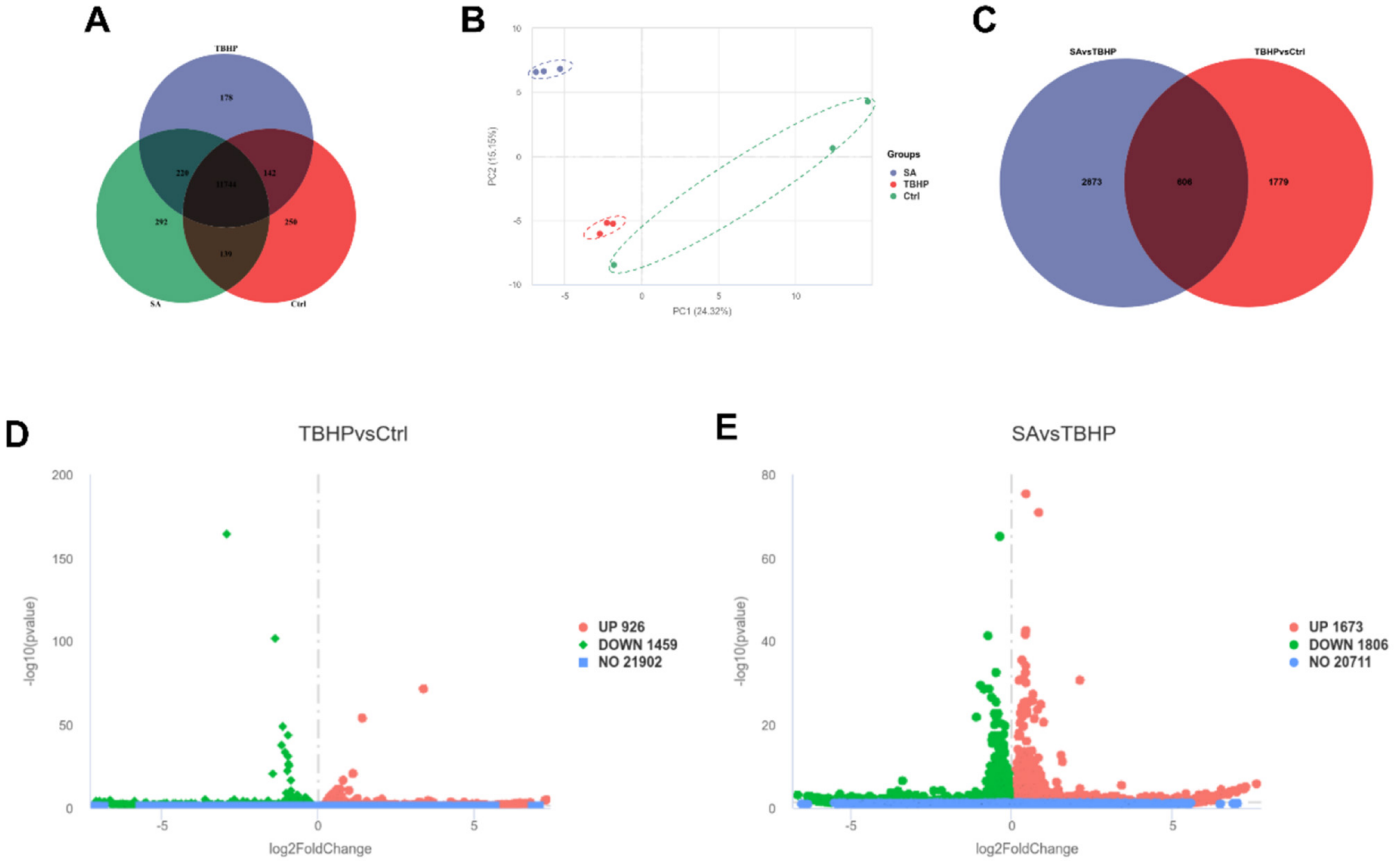
Differential gene expression analysis (*n* = 3). **(A)** Co-expression Venn plot of genes in Ctrl, TBHP, and SA groups. **(B)** Principal component analysis plot. **(C)** Venn plot of differentially expressed genes in Ctrl vs. TBHP and SA vs. TBHP groups. **(D)** Volcano plot of differentially expressed genes in Ctrl vs. TBHP group. **(E)** Volcano plot of differentially expressed genes in SA vs. TBHP group.

As demonstrated in the Co-expression Venn plot in Figure 4A, a total of 12,965 genes were identified by RNA-seq, of which 11,744 were shared by the Ctrl, TBHP, and SA groups. The results of the PCA are shown in Figure 4B, significant dispersion among the treatment groups, with good intra-group aggregation.

The identification of DEGs was conducted through the implementation of stringent filters, characterized by a |log_2_FoldChange| ≥ 1 and padj ≤ 0.05. As demonstrated in Figure 4C, a total of 3,479 DEGs were identified between the SA vs. TBHP groups, and 2,385 DEGs were detected between the TBHP vs. Ctrl groups, with 606 DEGs being shared among these comparisons. The volcano plots in Figures 4D, E demonstrate that TBHP treatment resulted in 926 DEGs that were found to be expressed at higher levels and 1,459 DEGs that were found to be expressed at lower levels in comparison with the Ctrl group, while SA treatment resulted in 1,673 DEGs that were found to be expressed at higher levels and 1,806 DEGs that were found to be expressed at lower levels in comparison with the TBHP group.

**Figure 5:**
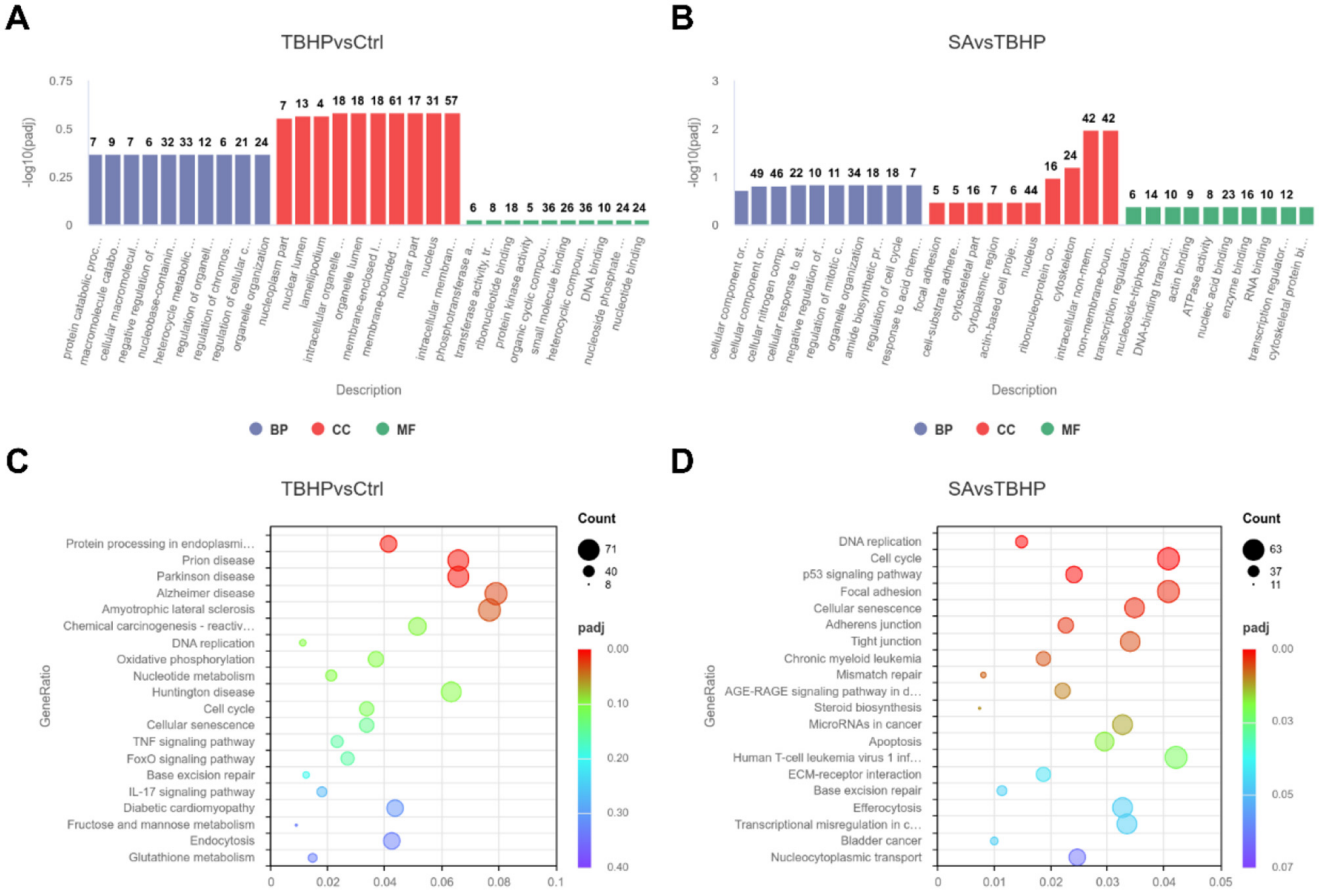
Functional enrichment analysis (*n* = 3). **(A)** Histogram of GO enrichment analysis for Ctrl vs. TBHP group. **(B)** Histogram of GO enrichment analysis for SA vs. TBHP group. **(C)** KEGG enrichment scatter plot for Ctrl vs. TBHP group. **(D)** KEGG enrichment scatter plot for SA vs. TBHP group.

GO function enrichment analysis of DEGs in the TBHP and Ctrl groups yielded 951 biological process (BP) terms, 145 molecular function (MF) terms, and 158 cellular component (CC) terms. As demonstrated in Figure 5A, the top 10 terms were selected to generate a GO function histogram. KEGG pathway enrichment analysis identified 325 pathways. As demonstrated in Figure 5C, the top 20 pathways, ranked by gene number, were utilized to generate KEGG classification scatter plots. The GO functional enrichment analysis of the SA and TBHP groups yielded 1,017 BP terms, 144 MF terms, and 160 CC terms. The top 10 terms were selected to generate the GO functional histogram (see Figure 5B). KEGG pathway enrichment analysis revealed 332 pathways. The top 20 pathways, sorted by gene number, were then used to generate KEGG classification scatter plots (see Figure 5D). The KEGG pathway enrichment analysis revealed pathways common to both comparison groups, including base excision repair, DNA replication, cell cycle, and cellular senescence.

### 3.5 Effect of shikimic acid on XRCC1, PARP1 protein expression and PAR levels

**Figure 6:**
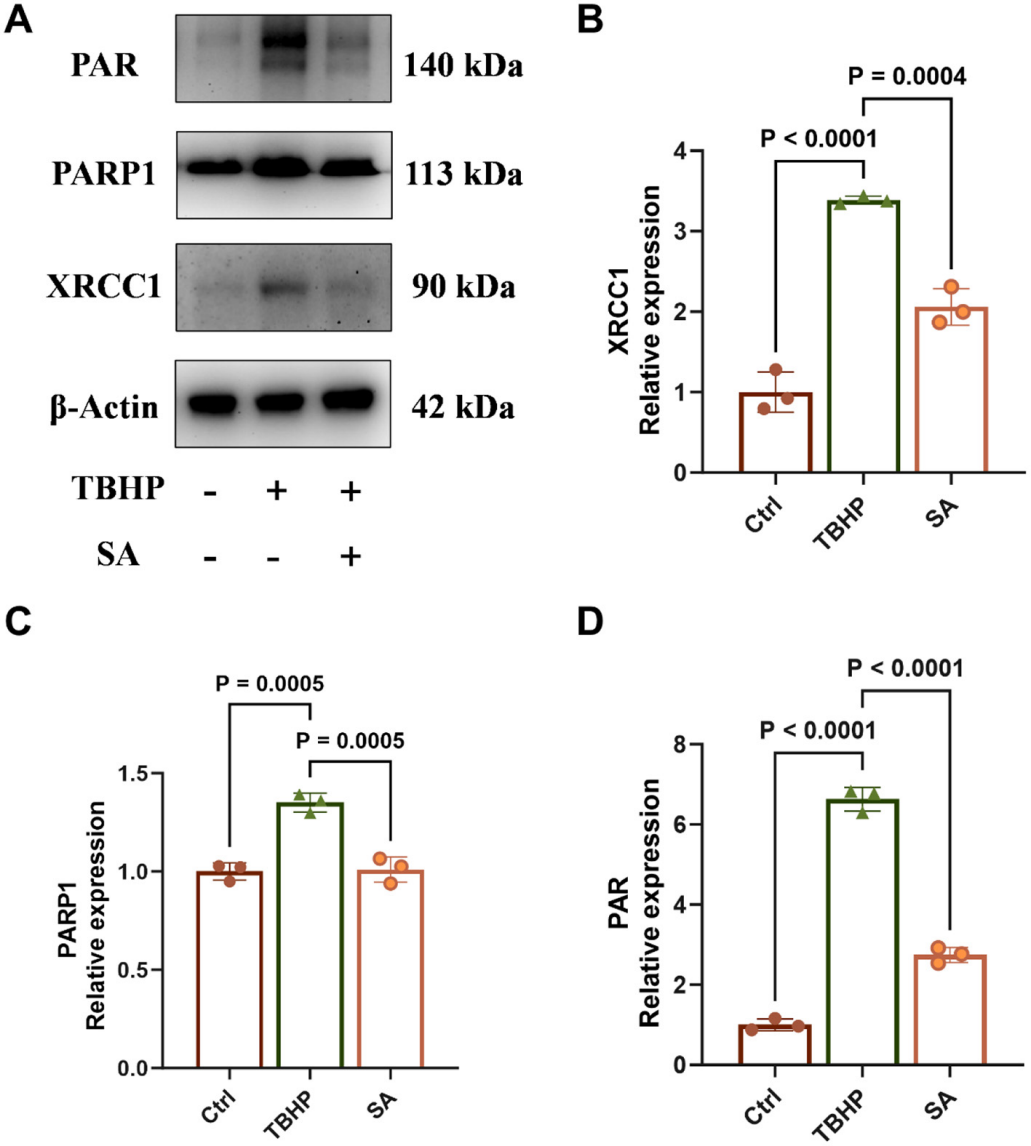
Effect of shikimic acid on XRCC1, PARP1 protein expression and PAR levels (*n* = 3, where 3 is the number of biological replicates per group). **(A)** Representative Western blot images for XRCC1, PARP1, and PAR levels. **(B)** Effect of SA on XRCC1 protein expression in 3D4/21 cells. **(C)** Effect of SA on PARP1 protein expression in 3D4/21 cells. **(D)** Effect of SA on 3D4/21 cells PAR levels.

TBHP treatment significantly increased the protein expression of XRCC1 and PARP1, and elevated PAR levels (indicative of PARP activity), compared to the control group (*P* < 0.05, Figures 6A–D). Conversely, SA treatment led to a significant decrease in the protein expression of XRCC1 and PARP1, and a decrease in PAR levels (*P* < 0.05).

### 3.6 Effect of shikimic acid on SASP factor production

**Figure 7:**
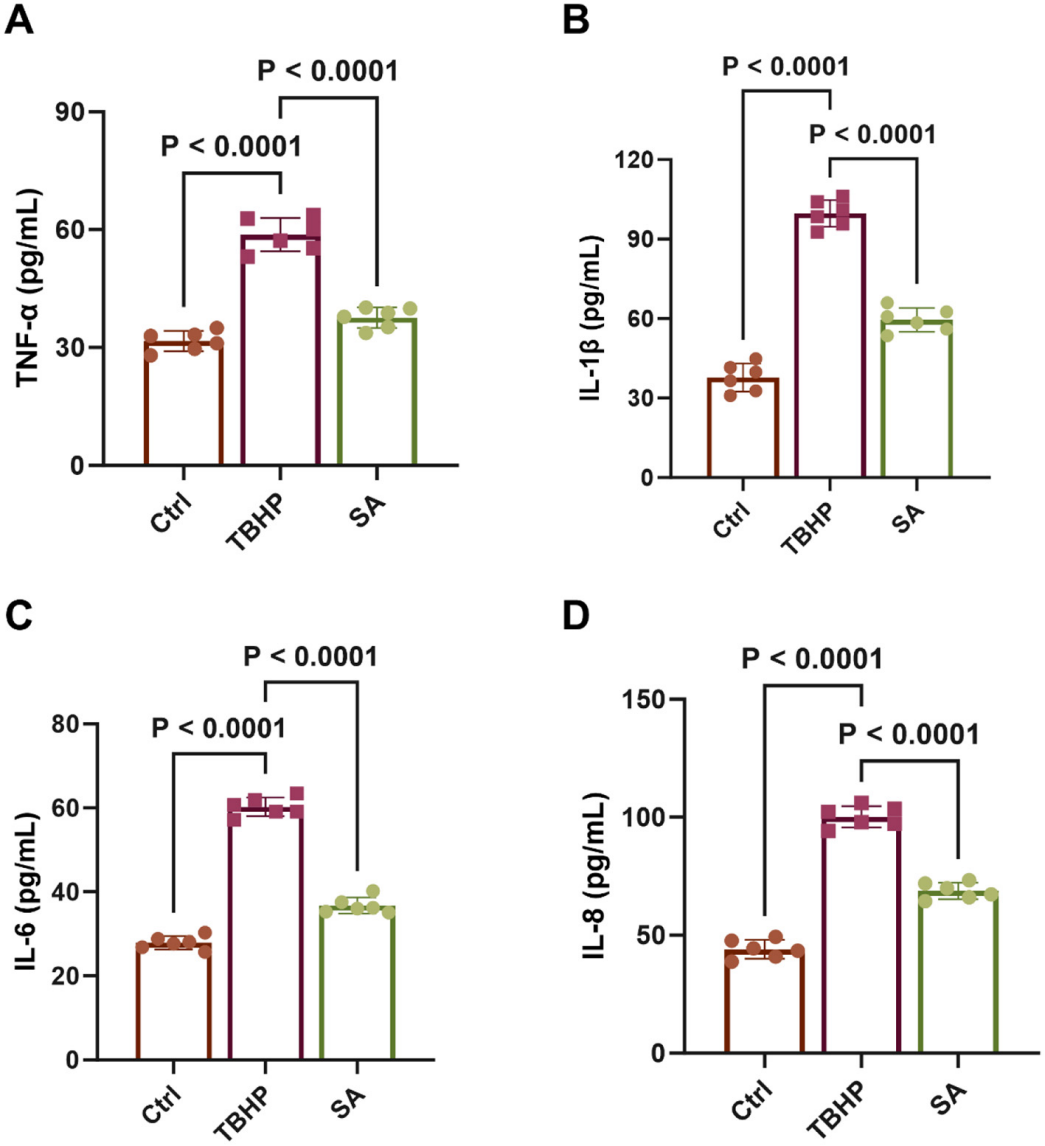
Effect of shikimic acid on SASP factor production (*n* = 6). **(A)** Effect of SA on TNF-α content in 3D4/21 cells. **(B)** Effect of SA on IL-1β content in 3D4/21 cells. **(C)** Effect of SA on IL-6 content in 3D4/21 cells. **(D)** Effect of SA on IL-8 content in 3D4/21 cells.

As demonstrated in Figure 7, the levels of TNF-α, IL-1β, IL-6, and IL-8 were significantly elevated in the medium of 3D4/21 cells in the TBHP group in comparison with the control group (*P* < 0.05). However, treatment with SA significantly reduced the levels of these specific SASP factors (*P* < 0.05).

### 3.7 Effect of shikimic acid on senescence of 3D4/21 CELLS

**Figure 8:**
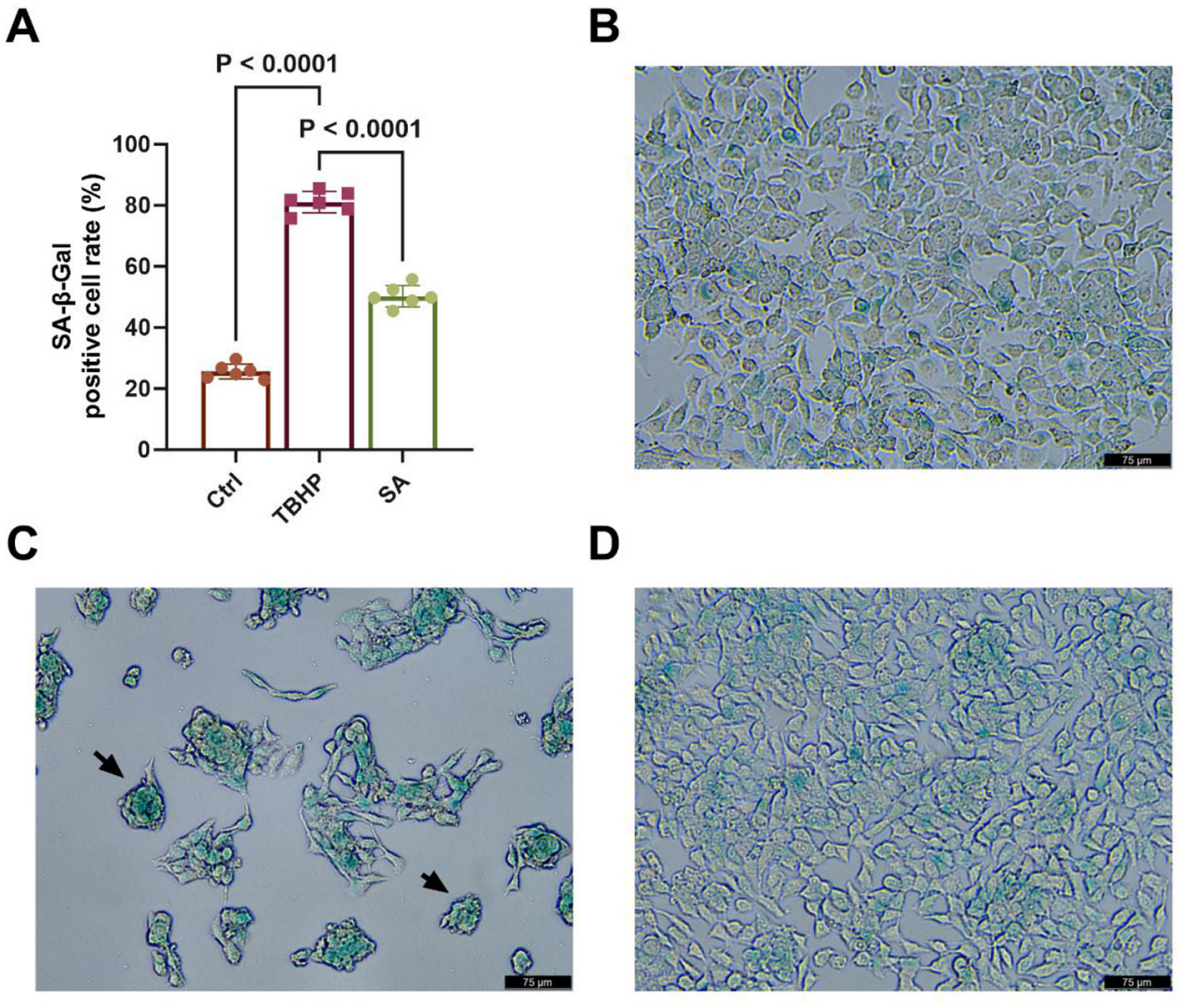
Effect of shikimic acid on senescence of 3D4/21 cells (*n* = 6). **(A)** Histogram of SA-β-gal-positive cell rate in Ctrl, TBHP, and SA groups. **(B)** Representative SA-β-gal staining image of the Ctrl group. **(C)** Representative SA- β -gal staining image of the TBHP group. **(D)** Representative SA-β-gal staining image of the SA group. Scale bar = 75 μm, 200 × magnification.

As demonstrated in Figure 8A, the proportion of SA-β-gal-positive cells in the TBHP group (approximately 81.12%) was considerably higher than that in the Ctrl group (approximately 25.63%) (*P* < 0.05). Conversely, the percentage of SA-β-gal-positive cells in the SA group was significantly lower than that in the TBHP group, at approximately 50.32% (*P* < 0.05).

As illustrated in Figures 8B–D, typical SA-β-gal staining image of 3D4/21 cells is evident in the Ctrl, TBHP and SA groups, respectively. As indicated by the black arrow in Figure 8C, positive cells exhibited characteristic cytoplasmic green staining and a rounded cell morphology.

## 4 Discussion

The present investigation reveals that SA exerts a significant protective effect on 3D4/21 cells against TBHP-induced oxidative stress-related toxicity, effectively restoring cellular homeostasis. Notably, the 3D4/21 cell line, an immortalized porcine AM line, preserves the essential structural features and biological functions of primary porcine AMs, thus validating its use as a model for *in vitro* investigations (16). While this model provides valuable insights and shares similarities with primary AMs, results should be interpreted with caution regarding direct extrapolation to primary human AMs or the complex *in vivo* lung microenvironment. Our findings demonstrate that SA enhances cell viability, effectively reduces key markers of oxidative stress (ROS, LDH) and inflammation (NO, iNOS, COX-2), modulates the DDR, particularly PARP activation, and markedly attenuates cellular senescence and SASP production.

The utilization of TBHP effectively induced an oxidative stress milieu, resulting in macrophage injury and inflammation (17), which are hallmarks of human lung diseases, where the management of macrophage-driven inflammation and redox imbalance is of the utmost importance (18). Importantly, SA pretreatment dose-dependently counteracted these detrimental effects. The observed decreases in ROS, NO, iNOS, and COX-2 levels validate SA's substantial antioxidant and anti-inflammatory capabilities in this context, corroborating earlier reports (11, 12). This suggests SA may act by directly neutralizing ROS and/or by bolstering cellular antioxidant defenses in AMs. The efficacy of 250 μM SA was often comparable to the well-established antioxidant NAC (e.g., for cell viability, LDH, and iNOS levels) and, in some cases, statistically superior (e.g., for reduction of ROS levels), highlighting its potent protective capabilities (19).

The protective profile of SA resonates with effects observed for other plant-derived polyphenols and organic acids known to combat oxidative stress and inflammation (20), such as resveratrol, quercetin, and chlorogenic acid (21–23). For instance, similar to SA, resveratrol has been shown to protect lung cells from oxidative damage and modulate inflammatory pathways (24). Quercetin has also been shown to exhibit potent antioxidant effects and can influence DNA repair and senescence (25). While broad antioxidant effects are common among these phytochemicals, SA's pronounced effect on normalizing PARP1 activation (PAR levels) and associated protein expression (PARP1, XRCC1) suggests a significant role in modulating the intensity of the DDR signaling cascade in macrophages. SA appears to effectively ‘reset' the hyperactive PARP1 response triggered by oxidative damage, potentially offering a distinct advantage in preventing downstream pathological consequences like NAD+ depletion and senescence trigger persistence (26).

To gain a broader understanding of SA's mechanism, we employed transcriptomics. RNA-seq analysis revealed substantial alterations in the gene expression profile of 3D4/21 cells upon TBHP exposure, affecting thousands of genes. Crucially, SA pretreatment reversed a significant portion of these TBHP-induced changes. KEGG pathway enrichment analysis provided critical insights, identifying pathways related to DNA replication, cell cycle, cellular senescence, and notably, BER as commonly enriched among the differentially expressed genes in both TBHP vs. Ctrl and SA vs. TBHP comparisons. This strongly suggested that SA exerts its protective effects, at least in part, by modulating these fundamental cellular processes disrupted by oxidative stress. Notably, our transcriptomic analysis also hinted at SA's broader influence on cellular processes beyond direct DDR modulation. Pathways related to cell cycle regulation, the p53 signaling pathway, mismatch repair, and apoptosis, alongside metabolic pathways such as oxidative phosphorylation and nucleotide metabolism, were also enriched, suggesting multifaceted protective effects of SA, although these specific pathways were not the focus of subsequent validation in this study.

PARP1 is a key sensor of DNA breaks, including those arising directly from oxidative stress or during repair processes. Upon activation, PARP1 synthesizes PAR chains, creating a platform to recruit downstream factors, critically including the XRCC1 scaffold complex for subsequent repair steps involving polymerases and ligases (27). Consistent with a robust DDR, we observed that TBHP stress significantly increased protein levels of XRCC1 and PARP1, alongside a marked elevation in cellular PAR levels. The observed upregulation of PARP1 and XRCC1 protein levels following TBHP treatment likely represents an adaptive cellular response to increased DNA damage. DNA damage signaling pathways can activate transcription factors leading to increased expression of DDR genes, thereby enhancing the cell's capacity to detect lesions (PARP1) and assemble repair complexes (XRCC1) (28). The anti-PAR antibody used detects extensive PARylation, indicative of substantial PARP enzyme activation rather than baseline levels (29). Cellular PAR accumulation serves as a direct indicator of PARP enzymatic activity (30), and its elevation following TBHP exposure confirms substantial PARP activation due to significant DNA damage. Importantly, SA pretreatment effectively normalized the TBHP-induced increases in XRCC1, PARP1, and PAR levels. The normalization of PAR levels is particularly significant, suggesting that SA prevents PARP1 hyperactivation. This likely results from SA's antioxidant effects reducing the initial DNA damage load, thereby lessening the stimulus for sustained PARP1 activity. By preventing PARP1 hyperactivation, SA may conserve cellular NAD+ and ATP pools, mitigating energy crisis and cell dysfunction often associated with severe oxidative stress (31). This controlled modulation, rather than complete inhibition, of the DDR represents a nuanced protective mechanism, potentially preventing the detrimental signaling linked to senescence induction and chronic disease progression where DDR dysregulation is implicated (32, 33). The modulation of DNA repair responses without complete inhibition represents a nuanced approach that may be beneficial in clinical settings. While our data strongly suggest that SA's modulation of the DDR outcome is primarily an indirect consequence of its antioxidant effects reducing the initial DNA damage load, further studies would be necessary to definitively exclude potential direct interactions with DDR pathway components.

Cellular senescence is increasingly recognized as a driver of aging and chronic diseases, including those affecting the lungs (34, 35). Targeting senescent cells or preventing their formation is an active area of therapeutic development. Our finding that SA significantly reduces both a key senescence marker (SA-β-gal positivity) and the secretion of multiple pro-inflammatory SASP factors (TNF-α, IL-1β, IL-6, IL-8) is particularly relevant. By limiting macrophage senescence and the associated chronic inflammation, SA could potentially interrupt pathological feedback loops that sustain lung injury and fibrosis (36). It is noteworthy that the reduction in measured SASP factors (TNF-α, IL-1β, IL-6, IL-8) likely results from both the decreased number of senescent cells producing them and potentially SA's broader anti-inflammatory properties observed in this study (e.g., reduced iNOS/COX-2 expression).

Given the central role of macrophage dysfunction driven by oxidative stress and inflammation in debilitating human lung diseases like COPD, and IPF (37, 38), these findings strongly suggest that SA warrants further investigation as a potential therapeutic agent. Its natural origin from sources like star anise might enhance interest in its translational development (39), although rigorous safety and efficacy evaluations specifically for respiratory therapeutic applications remain essential.

This study has limitations, primarily its reliance on an *in vitro* model (3D4/21 cell line) and a single chemical stressor (TBHP). Future studies should validate these findings in primary AMs from relevant species and utilize other stressors pertinent to lung disease (e.g., cigarette smoke extract, particulate matter, pathogens). *In vivo* studies in models of oxidative lung injury are warranted to confirm the therapeutic potential of SA. Further mechanistic work could also explore SA's effects on other DNA repair pathways and pinpoint its upstream molecular targets. Nevertheless, our current data strongly support the protective role of SA in AMs facing oxidative insults, mediated significantly through the coordinated modulation of DNA repair and senescence pathways.

## 5 Conclusion

In conclusion, the present study demonstrates that SA effectively protects 3D4/21 cells from TBHP-induced oxidative injury through a multi-pronged mechanism encompassing the mitigation of oxidative stress and inflammation, the modulation and normalization of the DDR centered around PARP1 activation, and the significant attenuation of cellular senescence and the associated pro-inflammatory secretory phenotype. By restoring cellular homeostasis through these coordinated actions, SA demonstrates protective effects warranting further preclinical investigation to fully evaluate its potential for therapeutic application in human chronic lung diseases driven by oxidative stress and AM dysfunction.

## Data Availability

The original contributions presented in the study are publicly available. This data can be found here: https://www.ncbi.nlm.nih.gov/bioproject/PRJNA1252006 (Accession number: PRJNA1252006).
